# International expert consensus on managing elective hernia repair in patients with Crohn’s disease using the Delphi method

**DOI:** 10.1093/bjsopen/zrag172

**Published:** 2026-10-16

**Authors:** Hans Lovén, Frederik Helgstrand, Hans Friis-Andersen, Conrad Ballecer, Frederik Berrevoet, Marja Boermeester, Hanna de la Croix, Christine Gaarder, Ismail Gögenur, Nadia Henriksen, William Hope, Kamal Itani, Lars N. Jørgensen, Ferdinand Köckerling, Jan Roland Lambrecht, Marc Miserez, Ulf Petersson, Thomas Pinkney, Benjamin K Poulose, Michael Rosen, Jacob Rosenberg, David L Sanders, Dominic Slade, Björn Törnqvist, Thue Bisgaard

**Affiliations:** Center for Surgical Science, Zealand University Hospital, Køge, Denmark; Digestive Disease Center, Bispebjerg Hospital, University of Copenhagen, Copenhagen, Denmark; Center for Surgical Science, Zealand University Hospital, Køge, Denmark; Department of Gastrointestinal Surgery, Aarhus University Hospital, Aarhus University, Aarhus, Denmark; Department of Surgery, Dignity Health St. Joseph’s Hospital and Medical Center, Creighton University School of Medicine—Phoenix Division, Phoenix, Arizona, USA; Department for General and HPB Surgery and Liver Transplantation, Ghent University Hospital, Ghent, Belgium; Department of Surgery, Amsterdam University Medical Center, Amsterdam, the Netherlands; Department of Surgery, Sahlgrenska University Hospital, Gothenburg, Sweden; Department of Surgery, Inflammatory Bowel Disease Surgical Research Group, Institute of Clinical Sciences, Sahlgrenska Academy, University of Gothenburg, Gothenburg, Sweden; Department of Traumatology, Faculty of Medicine, Oslo University Hospital Ulleval, University of Oslo, Oslo, Norway; Center for Surgical Science, Zealand University Hospital, Køge, Denmark; Digestive Disease Center, Bispebjerg Hospital, University of Copenhagen, Copenhagen, Denmark; Department of Surgery, Novant Health New Hanover Regional Medical Center, Wilmington, North Carolina, USA; Department of Surgery, VA Boston Healthcare System, Boston, Massachusetts, USA; Department of Surgery, Boston University School of Medicine, Harvard Medical School, Boston, Massachusetts, USA; Digestive Disease Center, Bispebjerg Hospital, University of Copenhagen, Copenhagen, Denmark; Berlin Hernia Center, Vivantes Humboldt Hospital, Academic Teaching Hospital of Charité University Medicine, Berlin, Germany; Department of Gastroenterological Surgery, Sykehuset Innlandet Hospital Trust, Hamar, Norway; Department of Abdominal Surgery, University Hospitals KU Leuven, Leuven, Belgium; Department of Surgery, Faculty of Medicine, Skåne University Hospital Malmö, Lund University, Lund, Sweden; Academic Department of Surgery, University of Birmingham, Birmingham, UK; MPH Center for Abdominal Core Health, Department of Surgery, The Ohio State University, Wexner Medical Center, Columbus, Ohio, USA; Division of Gastrointestinal Surgery, Department of Surgery, Northwestern University Feinberg School of Medicine, Chicago, Illinois, USA; Department of Surgery, Herlev Hospital, University of Copenhagen, Copenhagen, Denmark; Academic Department of Abdominal Wall Surgery, Royal Devon University Foundation Healthcare Trust, North Devon District Hospital, Barnstaple, UK; Department of General Surgery, Salford Royal Hospitals NHS Foundation Trust, Salford, UK; Department of Clinical Sciences, Danderyd Hospital, Karolinska Institutet, Stockholm, Sweden; Center for Surgical Science, Zealand University Hospital, Køge, Denmark

**Keywords:** inflammatory bowel disease, surgical mesh, abdominal wall surgery, clinical decision-making

## Abstract

**Background:**

Patients with Crohn’s disease (CD) may be predisposed to mesh-related complications such as impaired wound healing and fistula formation following hernia repair. With limited evidence and no established guidelines for hernia repair in CD, this Delphi study aimed to establish a hernia expert consensus on elective inguinal, incisional, and parastomal hernia repair in patients with CD.

**Methods:**

A three-round Delphi process including 21 hernia experts from Europe and the USA was conducted between 24 April and 15 October 2025. The primary outcome of interest was a consensus ≥ 75% among participants. Near consensus was defined as ≥ 70% agreement. Statements were developed from participant comments and questionnaire responses. Experts evaluated statements on timing, surgical approach, and mesh selection (synthetic non-absorbable, synthetic absorbable, biosynthetic, biological, and non-mesh) across different CD subtypes in elective inguinal, incisional, and parastomal hernia repair. Patients were categorized into three CD subtypes, namely abdominal fistulizing Crohn's disease (FCD), isolated perianal FCD, and non-FCD, with each assessed in both active and non-active inflammatory disease states.

**Results:**

Consensus or near consensus was achieved for 12 of 20 items (60%) in Round 1, 17 of 33 items (52%) in Round 2, and 13 of 29 items (45%) in Round 3. Overall, 42 of 72 unique items reached consensus (34 items) or near consensus (8 items). It was suggested that elective hernia repair should be avoided in CD patients with active inflammation, regardless of abdominal FCD or non-FCD. In non-active CD, synthetic non-absorbable mesh was suggested for inguinal, incisional, and parastomal hernia repair. Standard mesh placement was recommended for inguinal hernias, whereas retromuscular or preperitoneal mesh placement was recommended for incisional and parastomal hernias. No consensus was reached to avoid elective repair in isolated perianal FCD with active inflammation. The use of non-mesh repair, synthetic absorbable, biological, or biosynthetic mesh was generally not recommended in the elective setting.

**Conclusions:**

Consensus was reached that CD with non-active inflammation does not require deviation from standard hernia repair principles provided that the mesh is positioned in extraperitoneal positions. However, patients with CD may have an increased perioperative risk and the surgical management should be guided by shared decision-making.

## Teaser

Evidence to guide elective hernia repair in patients with Crohn’s disease is limited. An international Delphi study was conducted among hernia experts to establish consensus on surgical decision-making across common clinical scenarios. Consensus supported avoiding elective repair in active disease, whereas standard mesh-based repair in extraperitoneal planes was considered appropriate in non-active Crohn’s disease.

## Introduction

Implantation of a hernia mesh induces a persistent local foreign body reaction involving proinflammatory mediators such as tumour necrosis factor-α, interleukin-1β, and interleukin-6^1^. The inflammatory response in Crohn's disease (CD) involves many of the same mediators, contributing to transmural inflammation and impaired wound healing^2^. This biological overlap may theoretically increase the risk of mesh-related complications such as enterocutaneous fistulas and non-healing wounds^3,4^.

Fistulizing Crohn’s disease (FCD) affects approximately 17–50% of patients with CD^5^. Perianal manifestations are most common, followed by enteroenteric, rectovaginal, and enterocutaneous fistulas^6–8^. Due to the pathophysiological characteristics of CD, including variable inflammatory activity, mesh implantation may be problematic, as outlined above.

Evidence for long-term outcomes after elective hernia repair in CD remains limited. For incisional hernia repair, a recent retrospective cohort study^4^ with 335 patients suggested an increased risk of mesh-related complications, particularly in patients with abdominal FCD (aFCD). However, that study did not report CD activity status, mesh position, or mesh type. For inguinal hernia repair, a small case-control study^9^ with 26 patients reported an increased risk of short-term (< 30 days) postoperative complications in patients with inflammatory bowel disease (IBD), whereas another large cohort study^10^ with 1072 patients reported low long-term mesh-related complication rates, unaffected by IBD subtype (CD or ulcerative colitis), history of fistulas, or surgical approach (laparoscopic or open). However, that latter study^10^ included only patients with perianal FCD (pFCD), and not those with aFCD. To date, no studies have specifically addressed outcomes after parastomal hernia repair in CD.

The present Delphi consensus study aimed to establish international expert consensus for three different types of hernia repair in patients with CD and did not address emergency hernia repair. It was hypothesized that consensus could be achieved for several aspects. The primary objective was to define recommendations for the timing, surgical approach, and use of mesh in the elective repair of inguinal, incisional and parastomal hernias, considering different CD subtypes and inflammatory states (active and non-active).

## Methods

### Study design

This study used a modified Delphi methodology^11,12^ to achieve expert consensus, based on the CREDES recommendations^13^ for conducting and reporting Delphi studies. The process was conducted as a web-based electronic survey between 24 April and 15 October 2025.

The study investigators identified experts in abdominal wall surgery through professional networks. Efforts were made to identify hernia surgeons with additional experience in the management of patients with IBD while also aiming for variation in age and geographic location. Approximately 30 candidates were identified through these networks and assessed against predefined eligibility criteria: authorship of ≥ 30 peer-reviewed publications on hernia surgery, contribution to one or more international hernia guidelines, or current or previous membership of a national or international hernia society steering committee. Twenty-four experts fulfilled at least one eligibility criterion and were invited to participate.

### Topics selection and Delphi methodology

The study investigators developed the Delphi questionnaires. A pilot round with five independent surgeons was conducted to assess clarity, logic, and flow, and minor revisions were made accordingly.

Free-text comments from participants were systematically reviewed and synthesized by the study investigators to generate statements for rounds 2 and 3. These were categorized into: general surgical principles, inguinal, incisional, and parastomal hernia repair. All questions were mandatory, preventing missing data on specific items. Participants were allowed to review and modify their responses within each survey round (rounds 1–3) and were not blinded to their own responses within each round. Responses could not be altered after final submission of a round. After rounds 1 and 2, anonymized summary feedback from the previous round was provided before the next round. In total, the panel evaluated 72 questions (54 Likert scale and 18 multiple choice) during the three Delphi rounds (*Fig. S1*).

Surgical strategies for the different CD subtypes and inflammatory activity states were addressed. Non-active disease was defined as the absence of clinical, biochemical, endoscopic, or radiological signs of inflammation (for example, a normal CD activity index and/or negative objective findings). Active disease was defined as the presence of any of these features. Patients were categorized into three subtypes: aFCD, isolated pFCD, and non-FCD. Each subtype was evaluated in both active and non-active inflammatory states. Inguinal hernias of any type or size were included. Incisional hernias classified as European Hernia Society (EHS) W2 (defect width 4–10 cm) or W3 (defect width >10 cm) and parastomal hernias with a defect diameter of 5–10 cm and >10 cm were eligible for inclusion^14^.

Questionnaire items included multiple-choice questions and five-point Likert scale statements (strongly agree, agree, neutral, disagree, strongly disagree). For Likert scale items, consensus was defined a priori as ≥ 75%^15,16^ of respondents indicating agreement (strongly agree, agree) or disagreement (strongly disagree, disagree). For multiple-choice questions, consensus was defined as ≥ 75% of participants selecting the same option. Near consensus was defined as 70–74% of respondents for both multiple-choice questions and Likert scale statements. For Likert scale statements reaching modest agreement (70–80%), the full distribution of responses (proportions agreeing, neutral, and disagreeing) were reported together with the median and interquartile range (i.q.r.) to reflect the strength of agreement and the degree of divergence within the panel. For calculation of the median and i.q.r., responses were coded from 1 (strongly agree) to 5 (strongly disagree). Items reaching near consensus or consensus were not re-evaluated in the subsequent rounds and were thus treated as conclusive statements. The Delphi process was limited to three rounds^17^.

Round 1 comprised typical elective clinical scenarios with multiple-choice questions (*Appendix S1*). This approach captured decision-making patterns within standard surgical scenarios, presented the range of surgical options, and assessed variability in responses. Thus, the questions addressed timing and surgical approach (open mesh, open non-mesh, laparoscopic, and robot assisted). Additional items focused on mesh type (synthetic non-absorbable, synthetic absorbable, biosynthetic, and biological), mesh porosity (small, medium, and large pore), mesh weight (light, medium, and heavy), and mesh placement (onlay, retromuscular/preperitoneal, preperitoneal, and intraperitoneal). These aspects were evaluated for inguinal, incisional, and parastomal hernia repair, respectively (*Appendix S2*). Participants received three reminders per round, with approximately 1–3 months between rounds. Response rates were documented for each round, and all non-responders were reinvited for the following round to avoid selection bias.

### Outcomes of interest

The primary outcome of interest was a consensus ≥ 75%. Statement analysis was conducted according to the following categories: general surgical principles, inguinal hernia repair, incisional hernia repair, and parastomal hernia repair.

### Statistical analysis

Likert scale responses were treated as ordinal data and summarized as median and i.q.r., as described above. Categorical data were reported as numbers and percentages when relevant. All analyses were descriptive using standard summary statistics^18^.

## Results

### Panel

Of the 24 invited experts, 21 participated and 20 completed all three rounds. To minimize bias, the study investigators did not participate in the survey. Gastroenterologists and IBD surgeons without a special interest in abdominal wall surgery were not invited.

The panel included experts in abdominal wall surgery with expertise in colorectal surgery, IBD, intestinal failure, enterocutaneous fistulas, and surgical infections. Eight of 21 participants (38%) had a dedicated subspecialty practice in colorectal surgery, IBD, and/or intestinal failure. Most participants were active surgeons (90%) with access to robotic surgery (90%), and 67% were involved in the routine clinical management of CD. Full participant characteristics are presented in *Table S1*. In all, 42 of 72 unique items were classified as recommendations, comprising 34 consensus items and eight near-consensus items (*Table 1*).

**Table 1:** Items reaching consensus or near consensus regarding inguinal, incisional, and parastomal hernia repair in patients with CD

	Agreement round 1	Agreement round 2	Agreement round 3	Final agreement
**General principles**				
Active CD is defined as < 30 days from hernia repair			75%*	75%
Inform all CD patients about increased risk of postoperative complications such as infection, poor wound healing, or fistula formation		75%		75%
Patients with indeterminate colitis (that is, not sure if CD or UC) should be managed as if they have CD as a precautionary measure			85%	85%
Coordinate elective hernia repair (incisional and parastomal) with planned IBD surgery if possible		70%		70%
Use objective assessment (for example, faecal calprotectin, MRI, endoscopy, capsule endoscopy) when CD activity is uncertain			100%	100%
Avoid elective hernia surgery in aFCD with active inflammation		85%		85%
Avoid elective hernia surgery in non-FCD with active inflammation		70%		70%
**Inguinal hernia repair in non-active CD (all subtypes)**				
Surgical technique				
Use the same inguinal hernia repair technique in non-active aFCD and non-FCD	100%			100%
Use the same inguinal hernia repair technique in non-active aFCD and pFCD	85%			85%
For primary inguinal hernia repair in non-active CD, a minimally invasive approach (TEP/TAPP) should be the primary choice			70%	70%
Laparoscopic approach (both robotic and non-robotic)			75%*	75%
Use mesh				
Yes	95%			95%
No				
Mesh type				
Synthetic non-absorbable	85%			85%
Mesh weight				
Light or medium weight for Lichtenstein			85%	85%
Medium or heavy weight for TAPP/TEP			70%	70%
Mesh pore size				
Large pore		75%		75%
Other				
Avoid non-absorbable suture for closing of the peritoneum in TAPP		80%		75%
Non-active CD (all subtypes) is a risk factor for postoperative complications after inguinal hernia repair			80% disagree	80%
**Incisional hernia repair in non-active CD (all subtypes)**				
Surgical technique				
Use the same incisional hernia repair technique in non-active aFCD and non-FCD	100%			100%
Use the same incisional hernia repair technique in non-active aFCD and pFCD	92%			92%
For incisional hernia repair in non-active CD, open surgery carries a higher postoperative complication risk than minimally invasive surgery			75%	75%
Open and minimally invasive approaches using retromuscular mesh placement are equally safe with respect to postoperative complications and hernia recurrence in elective incisional hernia repair in non-active CD in general			70%	70%
Use mesh				
Yes	95%			95%
No				
Mesh type				
Synthetic non-absorbable	75%			75%
Mesh weight				
Medium weight		75%		75%
Mesh pore size				
Large pore		90%		90%
Mesh placement				
Retromuscular/preperitoneal	100%			100%
Other				
Closure of the posterior sheath is important to minimize the risk of fistula formation		85%		85%
Closure of the posterior sheath should be performed with absorbable sutures		85%		85%
Use the same hernia repair technique in defects measuring 4–10 and > 10 cm		75%		75%
Active CD (all subtypes) is a risk factor for postoperative complications after incisional hernia repair		90%		90%
Non-active CD (all subtypes) is a risk factor for postoperative complications after incisional hernia repair			75%	75%
**Parastomal hernia repair in non-active CD (all subtypes)**				
Surgical technique				
Use the same parastomal hernia repair technique in non-active aFCD and non-FCD	77%			77%
Use the same parastomal hernia repair technique in non-active aFCD and pFCD	85%			85%
Use mesh				
Yes	90%			90%
No				
Mesh type				
Synthetic non-absorbable		70%		70%
Mesh weight				
Medium		80%		80%
Mesh pore size				
Large pore		95%		95%
Mesh placement				
Retromuscular/preperitoneal		85%		85%
Other				
Active CD (all subtypes) is a risk factor for postoperative complications after parastomal hernia repair		95%		95%
Non-active CD (all subtypes) is a risk factor for postoperative complications after parastomal hernia repair			70%	70%
For parastomal hernia repair in non-active CD, mesh type and placement should be the same for defects 5–10 and > 10 cm			70%	70%

^*^Combined agreement. Near consensus was defined as 70–74% agreement, consensus was defined as 75–80% agreement, and strong consensus was defined as 81–100% consensus. CD, Crohn’s disease; UC, ulcerative colitis; IBD, inflammatory bowel disease; MRI, magnetic resonance imaging; aFCD, abdominal fistulizing Crohn’s disease; FCD, fistulizing Crohn’s disease (that is, both aFCD and pFCD); pFCD, isolated perianal fistulizing Crohn’s disease; TEP, totally extraperitoneal repair; TAPP, transabdominal preperitoneal repair.

### General surgical principles

In patients with active inflammation within 30 days before hernia repair, a trend was reported to avoid elective hernia repair in aFCD (85% agreement) and non-FCD (70% agreement, 0% neutral, 30% disagreement; median 2 (i.q.r. 1.5–4). There was no consensus to avoid elective repair in active pFCD (47% agreement). In the event of uncertainty regarding inflammatory activity, additional imaging or endoscopic assessment was recommended (100% agreement). It was recommended that patients with indeterminate colitis (that is, no clear differentiation between CD and ulcerative colitis) were managed as if they had CD (85% agreement). In non-active CD, mesh repair was recommended across all three hernia types (90–95% agreement), whereas non-mesh repair showed strong disagreement (5–10% agreement). The panel agreed that patients with CD without active inflammation could be managed uniformly within each type of hernia (77–100% agreement). There was a moderate agreement (75% agreement, 10% neutral, 15% disagreement; median 1.5 (i.q.r. 1–2.5)) that all patients with CD should be informed about the increased postoperative risks, such as wound infection, impaired wound healing, and fistula formation. Coordination of incisional and parastomal hernia repair with planned CD-related surgery was suggested when feasible. This reached near consensus (70% agreement, 5% neutral, and 25% disagreement; median 2 (i.q.r. 1–3.5); *Table 1*). Recommended surgical management and mesh use for patients with non-active CD undergoing inguinal, incisional, and parastomal hernia repair are shown in *Fig. 1*.

**Figure 1:**
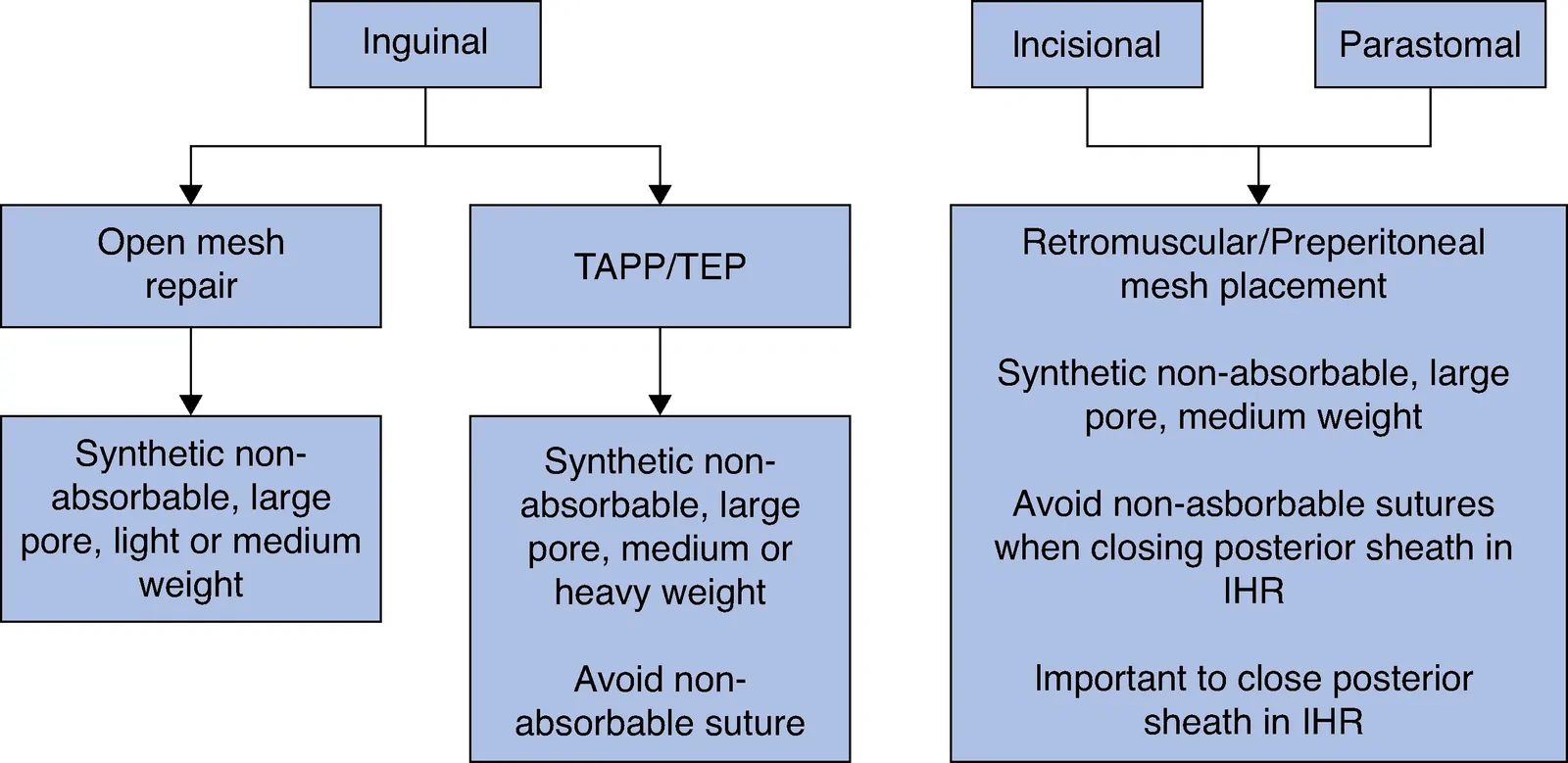
Treatment algorithm for elective inguinal, incisional, and parastomal hernia repair in patients with Crohn’s disease based on experts’ recommendations TAPP, transabdominal preperitoneal repair; TEP, totally extraperitoneal repair; IHR, incisional hernia repair.

### Inguinal hernia repair

A laparoscopic approach, either conventional or robot assisted, was suggested as the first choice for elective primary inguinal hernia repair in CD with non-active inflammation (75% agreement). Synthetic, non-absorbable, large-pore mesh was recommended for both open and laparoscopic repair (85% agreement). Light- or medium-weight mesh was recommended (85% agreement) for open repair. For laparoscopic repair, including transabdominal preperitoneal (TAPP) and totally extraperitoneal (TEP) approaches, there was near consensus in favour of medium- or heavy-weight mesh (70% agreement, 10% neutral, 20% disagreement; median 2 (i.q.r. 1.5–3)). The use of non-mesh repair, biological mesh, biosynthetic mesh, and absorbable synthetic mesh was generally not recommended (agreement 0–10%). Non-active CD was not considered a risk factor for postoperative complications following inguinal hernia repair (80% agreement, 5% neutral, 15% disagreement; median 4 (i.q.r. 4–4); *Table 1*).

### Incisional hernia repair

For elective incisional mesh repair in CD with non-active inflammation, it was suggested to use a non-absorbable (75% agreement), large-pore (90% agreement), medium-weight (75% agreement) synthetic mesh. In addition, the experts recommended retromuscular or preperitoneal mesh placement (100% agreement), regardless of laparoscopic or open access. The use of non-mesh repair, biological mesh, biosynthetic mesh, and absorbable synthetic mesh was generally not recommended (5–10% agreement). The use of absorbable sutures for posterior rectus sheath closure was recommended (85% agreement). It was suggested that defects measuring 4–10 and > 10 cm were managed using the same technique (70% agreement, 5% neutral, 25% disagreement; median 2 (i.q.r. 2–3.5)). Open mesh repair was considered to carry a higher postoperative risk than laparoscopic approaches (75% agreement, 5% neutral, 20% disagreement; median 2 (i.q.r. 2–2.5)). Nevertheless, near consensus indicated that both approaches were similarly safe regarding postoperative complications and recurrence (70% agreement, 5% neutral, 25% disagreement; median 2 (i.q.r. 2–3.5)). CD with both active and non-active inflammatory disease states was regarded as a risk factor for postoperative complications (90% and 75% agreement, 5% neutral, 20% disagreement; median 2 (i.q.r. 2–2.5, respectively); *Table 1*).

### Parastomal hernia repair

For elective parastomal hernia mesh repair in CD with non-active inflammation, retromuscular or preperitoneal mesh placement (85% agreement) with non-absorbable (70% agreement), large-pore (95% agreement), medium-weight (80% agreement) synthetic mesh was agreed (regardless of laparoscopic or open access). The use of non-mesh repair, biological mesh, biosynthetic mesh, and absorbable synthetic mesh was generally not recommended (5–10% agreement). It was suggested that defects measuring 5–10 and > 10 cm were managed with the same surgical technique (agree 70%, neutral 5%, disagree 25%; median 2 (i.q.r. 2–3.5)). Active and non-active inflammatory CD were both considered risk factors for postoperative complications. Consensus was reached for active disease (95% agreement), whereas non-active disease reached near consensus (70% agreement, 10% neutral, 20% disagreement; median 2 (i.q.r. 2–3); *Table 1*).

## Discussion

In the present Delphi analysis, it was suggested elective hernia surgery is avoided in aFCD and non-FCD with active inflammatory disease. In patients with CD and non-active inflammation, mesh repair was recommended for elective hernia repair. Non-mesh repair and the use of biological, biosynthetic, or synthetic absorbable mesh were generally not recommended in the elective setting. Synthetic non-absorbable mesh was suggested for all three hernia types, with retromuscular or preperitoneal placement for incisional and parastomal repair. The panel agreed that the potentially increased risk of postoperative complications underlines the need for careful preoperative planning and shared decision-making.

Avoiding elective hernia repair in aFCD and non-FCD with active inflammation aligns with previous cohort studies^19–21^ and a systematic review^22^ identifying active CD as a risk factor for postoperative complications (that is, intra-abdominal abscess, anastomotic leakage, surgical site infection, and disease recurrence) following abdominal surgery. Moreover, the Delphi experts recommended preoperative objective assessment when the inflammatory CD status was uncertain, consistent with the mentioned risks associated with surgery in active CD^19,20,22^. It was recommended that indeterminate colitis be managed as CD. This subgroup accounts for approximately 10–15% of patients with IBD^23^, making this recommendation clinically relevant.

Implantation of a foreign body hernia mesh induces persistent local immune activation^1^ and may, in patients with CD, lead to both systemic and local responses (for example, impaired wound healing, sinus formation, and fistulation). Furthermore, impaired tissue healing and potential patient frailty in patients with CD may predispose to early and long-term postoperative complications. Nevertheless, these risks may be lower with extraperitoneal mesh placement^24^. In the present Delphi analysis, a permanent synthetic mesh was recommended for non-active CD across all three types of hernia. Accordingly, non-mesh repair and the use of biological, biosynthetic, or absorbable synthetic mesh were not recommended for elective hernia repair. In the general hernia literature, biological mesh has been associated with higher rates of recurrence and reoperation^25,26^, whereas biosynthetic mesh has shown lower recurrence rates in observational series^27,28^. In CD, one case-control study involving 114 patients undergoing incisional hernia repair identified the use of biological mesh as a risk factor for postoperative septic morbidity^29^.

Non-mesh repair in incisional hernias has been associated with similar rates of early postoperative complications compared with mesh repair, whereas long-term follow-up demonstrated higher recurrence rates in the non-mesh group^30,31^. Whether these findings apply to patients with CD has not been studied. Thus, in active CD requiring emergency surgery, non-mesh repair may be considered a pragmatic alternative despite an increased risk of recurrence^30,31^, although the evidence is weak. However, these considerations were not addressed in the present study.

No consensus was reached regarding avoiding elective hernia repair in active pFCD, consistent with a recent cohort study^4^ (335 patients) reporting fewer mesh-related complications after incisional hernia repair in patients with pFCD compared with aFCD. However, disease activity and mesh type/placement was not addressed in that study. In the present study, experts further recommended informing all patients with CD of an elevated postoperative risk, including impaired healing and fistula formation. This is in line with studies^3,21,22^ reporting poorer outcomes in patients with than without IBD.

For inguinal hernia repair, a laparoscopic approach (conventional or robot assisted) was suggested for primary hernias. CD without active inflammation was not considered a risk factor for postoperative complications, consistent with a recent cohort study^10^ reporting low mesh-related complication rates in general in patients with IBD. The use of synthetic, non-absorbable, large-pore mesh was recommended, in accordance with updated EHS groin hernia guidelines^32^.

Regarding incisional and parastomal hernia repair, both active and non-active CD were considered postoperative risk factors. Retromuscular or preperitoneal placement of a synthetic, non-absorbable, large-pore, medium-weight mesh was recommended for both hernia types. This likely reflects concerns regarding intraperitoneal mesh placement in CD and evidence suggesting fewer mesh-related complications with extraperitoneal techniques^24^. Given the limited evidence on parastomal hernia repair in CD and the potential for contact between the mesh and bowel, extraperitoneal techniques may be preferred.

The present study has several limitations. First, the Delphi methodology is based on personal preference and interpretation of the scarce evidence. No standardized information (for example, a systematic or structured literature review) was provided to participants, which may have led to variation in the interpretation of available evidence.

Second, the results may be influenced by panel composition, question phrasing, and the level at which consensus was defined (for example, 60% or 75%)^13,17^. Although international experts were invited, the final panel mainly comprised participants from high-income Western countries. This may limit the external validity and generalizability of the study results. In addition, gastroenterologists and IBD surgeons without expertise in abdominal wall surgery did not participate. This may be both a study strength and limitation, ensuring hernia-specific expertise while potentially excluding broader IBD perspectives. However, the process followed the CREDES principles for transparency^13^ and the high response rate minimized the effect of participant dropout (that is, attrition bias).

Third, the level of CD activity described in the scenarios may have been interpreted differently by participants. The scenarios did not distinguish between symptomatic and deep remission^33^, which may have influenced responses.

Fourth, the core scenarios were restricted to non-immunosuppressed patients to isolate the effects of disease activity and hernia-related factors. This limits generalizability, because many patients with CD receive immunomodulators, biologics, or corticosteroids. The two statements addressing this population, namely perioperative pausing of maintenance therapy and prolonged antibiotic prophylaxis, did not reach consensus (both 35% agreement), highlighting an important area for future research.

Fifth, the questionnaire structure allowed participants to revisit previous answers within each round (risk of recall bias). As a result, assessments were not blinded across hernia types within each round but remained blinded between rounds. In addition, free-text comments were transformed into structured statements by the study investigators, which may have introduced interpretation bias.

Sixth, the use of typical surgical scenarios in the first round may have limited applicability in more complex clinical situations. However, the statements developed in subsequent rounds were designed to capture a broader range of surgical decision-making. Finally, the Delphi analysis focused on elective surgery, with limited considerations of emergent hernia repair.

In conclusion, experts suggested that elective hernia repair was not performed in patients with aFCD and/or non-FCD in the presence of active inflammation. In non-active CD, hernia repair (that is, inguinal, incisional, and parastomal) can be managed according to standard principles using synthetic non-absorbable mesh implanted extraperitoneally. Non-mesh repair and biological, biosynthetic, or absorbable synthetic mesh were generally not recommended in elective hernia repair.

## Supplementary Material

zrag172_Supplementary_Data

## Data Availability

The data that support the findings of this study are available from the corresponding author upon reasonable request.
