# Supplementary material for: International expert consensus on managing elective hernia repair in patients with Crohn’s disease using the Delphi method
Source: BJS Open. 2026 Oct 16;10(5):zrag172. doi: 10.1093/bjsopen/zrag172 (PMC13675246; doi:10.1093/bjsopen/zrag172)
Supplement: Supplementary file 1 [file zrag172_supplementary_data.docx]

**International expert consensus on managing elective hernia repair in patients**

**with Crohn’s disease using the Delphi method**

Hans Lovén^a,b^, Frederik Helgstrand^a^, Hans Friis-Andersen^c^, Conrad Ballecer^d^, Frederik Berrevoet^e^, Marja Boermeester^f^, Hanna de la Croix^g^, Christine Gaarder^h^, Ismail Gögenur^a^, Nadia Henriksen^b^, William Hope^i^, Kamal Itani^j^, Lars Nannestad Jørgensen^b^, Ferdinand Köckerling^k^, Jan Roland Lambrecht^l^, Marc Miserez^m^, Ulf Petersson^n^, Thomas Pinkney^o^, Benjamin K Poulose^p^, Michael Rosen^q^, Jacob Rosenberg^r^, David L Sanders^s^, Dominic Slade^t^, Björn Törnqvist^u^, Thue Bisgaard^a^

^a^Center for Surgical Science, Zealand University Hospital, Køge, Denmark
^b^Digestive Disease Center, Bispebjerg Hospital, University of Copenhagen, Copenhagen, Denmark
^c^Department of Gastrointestinal Surgery, Aarhus University Hospital, Aarhus University, Aarhus, Denmark
^d^Department of Surgery, Dignity Health St. Joseph’s Hospital and Medical Center, Creighton University School of Medicine – Phoenix Division, Phoenix, AZ, USA
^e^Department for General and HPB Surgery and Liver Transplantation, Ghent University Hospital, Ghent, Belgium
^f^Department of Surgery, Amsterdam University Medical Center, Amsterdam, Netherlands
^g^Department of Surgery, Sahlgrenska University Hospital, University of Gothenburg, Institute of Clinical Science, Gothenburg, Sweden
^h^Department of Traumatology, Oslo University Hospital Ulleval, Faculty of Medicine, University of Oslo, Oslo, Norway
^i^Department of Surgery, Novant Health New Hanover Regional Medical Center, Wilmington, NC, USA
^j^Department of Surgery, VA Boston Healthcare System, Boston, Massachusetts; Boston University School of Medicine; Harvard Medical School, USA
^k^Berlin Hernia Center, Vivantes Humboldt Hospital, Academic Teaching Hospital of Charité University Medicine, Berlin, Germany
^l^Department of Gastroenterological Surgery, Sykehuset Innlandet Hospital Trust, Hamar, Norway
^m^Department of Abdominal Surgery, University Hospitals KU Leuven, Leuven, Belgium
^n^Department of Surgery, Skåne University Hospital Malmö, Faculty of Medicine, Lund University, Sweden
^o^Academic Department of Surgery, University of Birmingham, Birmingham, United Kingdom
^p^MPH Center for Abdominal Core Health, Department of Surgery, The Ohio State University, Wexner Medical Center, Columbus, OH, USA
^q^Division of Gastrointestinal Surgery, Department of Surgery, Northwestern University Feinberg School of Medicine, Chicago, IL, USA
^r^Department of Surgery, Herlev Hospital, University of Copenhagen, Copenhagen, Denmark
^s^Academic Department of Abdominal Wall Surgery, Royal Devon University Foundation Healthcare Trust, North Devon District Hospital, Barnstaple, United Kingdom
^t^Department of General Surgery, Salford Royal Hospitals NHS Foundation Trust, Salford, United Kingdom
^u^Department of Clinical Sciences, Danderyd Hospital, Karolinska Institutet, Stockholm, Sweden

**Corresponding author:** Hans Lovén, MD., Center for Surgical Science, Zealand University Hospital, Lykkebækvej 1, 4600 Køge, Denmark

**ORCID ID:** 0000-0002-7887-3579

**Supplementary Materials - Index**

| **Supplementary Appendices** |  |  |
| --- | --- | --- |
| Appendix 1. Patient scenarios |  | *page 3* |
| Appendix 2. All questions and statements |  | *page 4-9* |
|  |  |  |
| **Supplementary Figures and Tables** |  |  |
| Supplementary Table S1 Demographic and professional characteristics of the participating experts |  | *page 10* |
| Supplementary Figure S1 Total number of items reaching consensus or near-consensus per round |  | *page 11* |

**Supplementary Appendices**

Abbreviations:

BMI, Body mass index (kg/m²)

CD, Crohn’s disease
CDAI, Crohn’s disease activity index
FCD, abdominal fistulising Crohn’s disease
aFCD, abdominal fistulizing Crohn’s disease

pFCD, isolated perianal fistulizing Crohn’s disease
Non-FCD, non-fistulizing Crohn’s disease
TAPP, transabdominal preperitoneal repair
TEP, totally extraperitoneal repair
E-TEP, extended view totally extraperitoneal repair
MRI, magnetic resonance imaging
UC, ulcerative colitis

**Appendix 1. Patient scenarios**

Case 1. Inguinal hernia repair

A 45-year-old male with a BMI of 30 kg/m², diagnosed with abdominal fistulizing Crohn’s disease (aFCD) and a history of previous enterocutaneous fistulas. The patient underwent a laparoscopic ileocecal resection two years ago, with no signs of inflammation since (e.g., negative imaging, endoscopy and normal CDAI). Not on immunosuppressive IBD medication. The patient presents with a symptomatic left-sided inguinal hernia. No other abdominal surgery besides the one mentioned above. No adhesions or peritonitis described during the initial operation. The hernia significantly impacts quality of life, and an elective repair was offered.

How would you perform this elective inguinal hernia repair?

Case 2. Incisional hernia repair

A 45-year-old male with a BMI of 30 kg/m², diagnosed with abdominal fistulising Crohn’s disease (aFCD) and a history of previous enterocutaneous fistulas. Operated with an exploratory laparotomy with small bowel resection two years ago with no signs of inflammation since (e.g., negative imaging, endoscopy and normal CDAI). Not on immunosuppressive IBD medication. The patient presents with a symptomatic incisional hernia (5 cm horizontal and 6 cm vertical) in the earlier midline scar. There is no history of other abdominal operations than mentioned above. There were no adhesions or peritonitis described during the initial operation. The hernia has substantial impact on quality of life. The patient was offered a hernia repair.

How would you perform this elective incisional hernia repair?

Case 3. Parastomal hernia repair

A 45-year-old male with a BMI of 25 kg/m², diagnosed with abdominal fistulising Crohn’s disease (aFCD) and a history of previous enterocutaneous fistulas. Operated with an exploratory laparotomy with sigmoid resection and left-sided colostomy two years ago with no signs of inflammation since (e.g., negative imaging, endoscopy and normal CDAI). Not on immunosuppressive IBD medication. The patient presents with a symptomatic parastomal hernia (6 cm defect). There were no adhesions or peritonitis described during the initial operation. There is no history of other abdominal operations than mentioned above. It has been assessed that stoma reversal is not possible. The hernia has substantial negative impact on quality of life, and the patient was offered an elective repair.

How would you perform this elective parastomal hernia repair?

## **Appendix 2. Questions and statements for all three rounds** General surgical principles: Multiple choice questions for round 2 with distribution of answers (%) How should active CD be defined in the context of hernia surgery? Active at the time of the hernia surgery (30%) Active within the last 3 months (45%) Active within the last 6 months (15%) Active within the last 12 months (10%)

### Likert scale statements for round 2 and 3 with final agreement in parenthesis (%)

- Elective hernia surgery should be avoided in patients with active FCD (85%)
- Elective hernia surgery should be avoided in patients with active non-FCD (70%)
- All CD patients should be informed about an increased risk of postoperative complications (following the hernia repair) such as infection, poor wound healing or fistula formation (75%)
- Elective parastomal or incisional hernia repair should, if possible, be coordinated with any planned abdominal IBD surgery (70%)
- Before elective hernia repair in CD, remission should be confirmed through objective assessment, e.g. faecal calprotectin, MRI, endoscopy and/or capsule endoscopy (65%)
- Use objective assessment (e.g. faecal calprotectin, MRI, endoscopy and/or capsule endoscopy) when CD activity is uncertain (100%)
- Patients with indeterminate colitis should be managed as if they have CD, as a precautionary measure (85%)
- Elective hernia surgery should be avoided in patients with active isolated perianal FCD (50%)
- All non-active CD patients should be on prophylactic IBD medical therapy prior to elective hernia repair technique (60%)
- Elective parastomal and incisional hernia surgery should be avoided in patients with active perianal FCD (60%)
- In patients with non-active CD undergoing elective parastomal or incisional hernia repair, ongoing maintenance therapy (e.g. immunomodulators and biologics) should be paused perioperatively (35%)
- In non-active CD patients on maintenance immunosuppressive or biologic therapy, postoperative antibiotic prophylaxis should be prolonged after elective parastomal or incisional hernia repair (35%)
- Before elective hernia repair, CD patients should routinely undergo preoperative nutritional screening (e.g. albumin and key micronutrients) (50%)
- CD patients should receive additional ambulatory follow-up after elective hernia repair compared with non-CD patients (30%)

### Inguinal hernia repair: Multiple choice questions for round 1 (case 1) with distribution of answers (%) Surgical approach

Robot-assisted laparoscopic (TAPP or TEP) (5%)

Laparoscopic (TAPP or TEP) (45%)

Open mesh (e.g. Lichtenstein) (45%)

Open non-mesh (5%)

Use the same inguinal hernia repair technique in non-active aFCD and non-FCD (100%)

Use the same inguinal hernia repair technique in non-active aFCD and isolated perianal FCD (85%)

Mesh type

Synthetic non-absorbable (85%)

Synthetic absorbable (5%)
Biosynthetic (10%)

Biological (0%)

Mesh weight

Light (30%)

Medium (55%)

Heavy (15%)

Mesh porosity

Small pore (10%)
Medium pore (15%)

Large pore (75%)

Likert scale statements (for rounds 2 and 3) with final agreement in parenthesis (%)

- Synthetic non-absorbable mesh is preferable in patients with non-active CD (85%)
- When closing peritoneum in inguinal hernia TAPP repair, non-absorbable suture should be avoided in patients with CD (80%)
- Non-active CD (all subtypes: non-FCD, FCD and isolated perianal FCD) is a risk factor for postoperative complications after inguinal hernia repair (20%)
- Totally extraperitoneal repair (TEP) has a lower risk of mesh-related complications (such as fistulas interacting with the mesh) compared with transabdominal preperitoneal repair (TAPP) in patients with CD (active and non-active) (45%)
- Recurrence and complication risks are comparable between open Lichtenstein repair and minimally invasive approaches (TAPP/TEP) in elective inguinal hernia surgery for any CD subtype (i.e. non-FCD, FCD and isolated perianal FCD) (50%)
- Absorbable synthetic mesh is the preferred choice when operating patient with active CD (20%)
- Biological/biosynthetic mesh is the preferred choice when operating a patient with active CD (30%)
- Active CD (all subtypes: non-FCD, FCD and isolated perianal FCD) is a risk factor for postoperative complications after inguinal hernia repair (60%)
- Recurrence and complication risks are comparable between open Lichtenstein repair and minimally invasive approaches (TAPP/TEP) in elective inguinal hernia surgery for any CD subtype (i.e. non-FCD, FCD and isolated perianal FCD) (50%)
- Totally extraperitoneal repair (TEP) has a lower risk of mesh-related complications (such as fistulas interacting with the mesh) compared with transabdominal preperitoneal repair (TAPP) in patients with CD (active and non-active) (45%)
- Elective inguinal hernia surgery should be avoided in patients with active perianal FCD (55%)
- For synthetic non-absorbable mesh, light or medium weight mesh is preferable for TAPP and TEP (85%)
- For synthetic non-absorbable mesh, medium or heavy weight mesh is preferable for Lichtenstein repair (70%)
- For primary inguinal hernia repair in non-active CD, a minimally invasive approach (TEP/TAPP) should be the first-line choice (70%)
- When TAPP is not feasible (e.g. due to extensive intra-abdominal adhesions), TEP/E-TEP is preferable to open Lichtenstein for primary inguinal hernia repair in CD (25%)

### Incisional hernia repair: Multiple choice questions for round 1 (case 2) with distribution of answers (%) Surgical approach

Laparoscopic transabdominal (5%)

Laparoscopic E-TEP (0%)

Laparoscopic robot-assisted transabdominal (5%)

Laparoscopic robot-assisted E-TEP (25%)
Open mesh (60%)

Open non-mesh (5%)

Use same surgical approach for non-active aFCD and non-FCD (100%)

Use same surgical approach for non-active aFCD and isolated perianal-FCD (95%)

Mesh type

Synthetic non-absorbable (75%)

Synthetic absorbable (10%)
Biosynthetic (5%)

Biological (10%)

Mesh weight

Light (15%)

Medium (75%)

Heavy (10%)

Mesh porosity

Small pore (5%)
Medium pore (5%)

Large pore (90%)

Mesh placement

Retromuscular/preperitoneal (100%)

Preperitoneal (0%)

Intraperitoneal (0%)

Onlay (0%)

Likert scale statements (for rounds 2 and 3) with final agreement in parenthesis (%)

- Retromuscular mesh placement is preferable in incisional hernia repair for CD patients in general (90%)
- For incisional hernia repair in CD, posterior fascial closure is important to minimise the risk of fistula formation (85%)
- For incisional hernia repair in CD, closure of the posterior rectus sheath should be performed using absorbable sutures (85%)
- For incisional hernia repair in CD, mesh type and mesh placement should be the same for defects measuring 4–10 cm and >10 cm (75%)
- Open and minimally invasive approaches using retromuscular mesh placement are equally safe with respect to postoperative complications and hernia recurrence in elective incisional hernia repair in non-active CD (70%)
- Non-active CD (all subtypes: non-FCD, FCD and isolated perianal FCD) is a risk factor for postoperative complications after incisional hernia repair (75%)
- Active CD (all subtypes: non-FCD, FCD and isolated perianal FCD) is a risk factor for postoperative complications after incisional hernia repair (90%)
- Synthetic absorbable mesh is preferable in patients with active CD (35%)
- Biological or biosynthetic mesh is preferable in patients with active CD (35%)
- For incisional hernia repair in non-active CD, open surgery carries a higher postoperative complication risk than minimally invasive surgery (75%)
- When a transabdominal approach is not feasible (e.g. due to extensive intra-abdominal adhesions), TEP/E-TEP is preferable to an open approach for incisional hernia repair in CD in general (40%)
- For primary incisional hernia repair in non-active CD, a minimally invasive approach (TEP/E-TEP/TAPP/TARM) should be the primary choice (45%)

### Parastomal hernia repair: Multiple choice questions for round 1 (case 3) with distribution of answers (%) Surgical approach Laparoscopic approach (5%) Laparoscopic robot-assisted approach (30%) Open approach (laparotomy) with mesh (15%) Open local revision (no laparotomy) with mesh (40%) Open local revision (no laparotomy) without mesh (5%) Stoma on contralateral side (5%) Use same surgical approach for non-active aFCD and non-FCD (75%) Use same surgical approach for non-active aFCD and isolated perianal FCD (90%)

Mesh type

Synthetic non-absorbable (40%)

Synthetic funnel shaped non-absorbable (10%)

Synthetic absorbable (20%)
Biosynthetic (20%)

Biological (10%)

Mesh weight

Light (20%)

Medium (80%)

Heavy (0%)

Mesh porosity

Small pore (0%)
Medium pore (5%)

Large pore (95%)

Mesh placement

Onlay (15%)
Retromuscular/preperitoneal (using Pauli technique) (50%)

Retromuscular/preperitoneal (using keyhole technique) (30%)

Intraperitoneal (using Sugarbaker technique) (0%)

Intraperitoneal (using keyhole technique) (5%)

Intraperitoneal (using Sandwich technique) (0%)

Likert scale statements (for rounds 2 and 3) with final agreement in parenthesis (%)

- Retromuscular mesh placement is preferable in parastomal hernia repair for CD patients in general (85%)
- For patients with non-active CD (all subtypes: non-FCD, FCD and isolated perianal FCD) synthetic non-absorbable mesh is preferable (70%)
- Non-active CD (all subtypes: non-FCD, FCD and isolated perianal FCD) is a risk factor for postoperative complications after parastomal hernia repair (70%)
- For parastomal hernia repair in CD, mesh type and mesh placement should be the same for defects measuring 5–10 cm and >10 cm (70%)
- Open and minimally invasive approaches using retromuscular mesh placement are equally safe in terms of postoperative complications and hernia recurrence in parastomal hernia repair for patients with non-active CD (50%)
- Hybrid mesh (permanent synthetic component combined with an absorbable or biological part) is preferable for parastomal hernia repair in CD patients to avoid exposure of the lateralised bowel to synthetic mesh (30%)
- Active CD (all subtypes) is a risk factor for complications after parastomal hernia repair (95%)
- For patients with active CD, absorbable synthetic mesh is preferable (35%)
- For patients with active CD, biological or biosynthetic mesh is preferable (50%)
- For parastomal hernia repair in non-active CD, open surgery carries a higher postoperative complication risk than minimally invasive surgery (60%)
- When transabdominal approach is not feasible (e.g. due to extensive intra-abdominal adhesions), TEP/E-TEP is preferable to open approach for parastomal hernia repair in CD in general (20%)
- In non-active CD with primary parastomal hernia, the surgical approach should, when feasible, follow this preferred order: 1) stoma reversal, 2) open local revision + retromuscular mesh, 3) minimally invasive + retromuscular mesh, 4) open laparotomy + retromuscular mesh (50%)

**Supplementary Figures and Tables**

**Table S1. Demographic and professional characteristics of the participating experts**

| Category | Variable | n | % |
| --- | --- | --- | --- |
|  |  |  |  |
| Age | Median (range) | 55 (44–72) |  |
|  | Mean (SD) | 56.3 (8.3) |  |
| Nationality | US | 5 | 24% |
|  | Europe* | 16 | 76% |
| Active surgeon | Yes | 19 | 90% |
|  | No | 2 | 10% |
| Hospital beds | <20 | 0 | 0% |
|  | 20–40 | 4 | 19% |
|  | >40 | 17 | 81% |
| Daily involved in CD management | Yes | 14 | 67% |
|  | No | 7 | 33% |
| Access to robot assisted surgery? | Yes | 19 | 90% |
|  | No | 2 | 10% |
| Participated in all three rounds | Yes | 20 | 95% |
|  | No | 1 | 5% |

*Belgium (n=2), Denmark (n=4), Germany (n=1), Netherlands (n=1), Norway (n=2), Sweden (n=3), United Kingdom (n=3)

**Figure S1. Total number of questions/statements reaching consensus or near-consensus per round**

Round 1 used multiple-choice questions and rounds 2 and 3 used Likert-scale statements. Statements not reaching consensus or near-consensus in round 2 were re-assessed in round 3 and counted in both rounds, so per-round totals reflect items assessed per round, not unique items.
